# Longitudinal Follow-up Survey of Effects of Oral Comprehensive Healthcare Measures on Early Childhood Caries

**DOI:** 10.3290/j.ohpd.a43347

**Published:** 2020-02-12

**Authors:** Ting Cui, Quanchen Xu, Yili Wu, Xiaoxiao Yang, Ting Li, Huibin Sun

**Affiliations:** a Master’s Degree Candidate, Doctor, Department of Stomatology, The Affiliated Hospital of Qingdao University, Qingdao, China. Design of the study, questionnaires and draft manuscripts and the preparation of the questionnaire; responsible for the oral health examination and oral health education; finalising the manuscript.; b Associate Professor, Doctor, Department of Stomatology, The Affiliated Hospital of Qingdao University, Qingdao, China. Responsible for the analysis of the data and summary of the results.; c Associate Professor, Department of Epidemiology and Biostatistics, Medical College, Qingdao University, Qingdao, China. Responsible for the analysis of the data and summary of the results.; d Master’s Degree Candidate, Doctor, Department of Stomatology, The Affiliated Hospital of Qingdao University, Qingdao, China. Responsible for the oral health examination and oral health education.; e Master’s Degree Candidate, Doctor, Department of Stomatology, The Affiliated Hospital of Qingdao University, Qingdao, China. Responsible for the oral health examination and oral health education.; f Associate Professor, Doctor, The Affiliated Hospital of Qingdao University, Qingdao, China. Directed and designed the study, questionnaires and draft manuscripts and the preparation of the questionnaire; finalising the manuscript.

**Keywords:** early childhood caries (ECC), child, oral hygiene behaviour, comprehensive oral healthcare, oral health education

## Abstract

**Purpose:**

To assess the effect of oral comprehensive healthcare on the prevalence of early childhood caries in urban areas of China.

**Materials and Methods:**

A total of 398 children aged 4–5 years from six different kindergartens in Qingdao were recruited to participate in a 1-year single-blind randomised controlled clinical trial. They were randomly assigned into either an experimental group (187 children) or a control group (211 children). The experimental group received comprehensive oral healthcare including oral health examinations, oral health education for children and their guardians, and local fluoride application and dental treatment, whereas children in the control group only received oral health examinations twice a year. The children’s parents finished a comprehensive oral health questionnaire before and after the experiment. After a year, the oral health status of two groups of children was statistically analysed to determine the effect of oral comprehensive healthcare on early childhood caries.

**Results:**

After 1 year, the numbers of decayed teeth (dt), decayed tooth surfaces (ds), filled teeth (ft), and filled tooth surfaces (fs) in the experimental group were significantly lower than the control group (p <0.001). The dmft and dmfs were also significantly different between the two groups (p <0.05). Statistically significant differences were additionally shown in oral hygiene habits and eating habits of both the children and their parents in the two groups compared with 1 year before (all p <0.001).

**Conclusion:**

Implementation of comprehensive oral healthcare measures both prevents and reduces early childhood caries.

Early childhood caries (ECC) is a common and complex bacterial disease. Affected teeth may be secondary to pulpitis and apical periodontitis, but also can cause inflammation of the entire jaw.^2,6^ If not treated in time, caries develops rapidly and can lead to loss of teeth. ECC and its associated complications can also affect the health of the whole body.^4^ ECC has become a common disease that affects children’s health. In the third Chinese national oral health epidemiological survey in 2005, the prevalence rate of caries in children aged 5 years was 65.1%.^30^ In the fourth national oral health epidemiology survey in 2015, the prevalence rate of caries in children aged 5 years in China was 70.9%, which rose by 5.8 percentage points compared to what it was 10 years ago.^19^ The prevalence of ECC is on the rise, but forming good oral health behaviour remains a powerful measure for preventing and reducing caries. Dental caries is typically evaluated according to the International Caries Classification and Management System (ICDAS) assessment.^22^ Studies have shown that the oral health concept of parents can directly affect the oral health behaviour of their children.^9,29^

ECC was defined by the American Academy of Pediatric Dentistry (AAPD) in 1999 as one or more carious teeth (no cavitation), lost teeth for caries or filled teeth on any tooth surface of deciduous teeth in the oral cavity of children aged 71 months or younger.^7^ AAPD has also defined children under the age of 3 who have one or more surface caries of smooth teeth, lost teeth because of caries, or teeth filled for caries as S-ECC.^5^ Caries tends to develop rapidly in children and is difficult to control. In the 2015 fourth oral health survey, only 24.1% of children aged 5 brushed teeth twice a day, and only 42.1% of them used fluoridated toothpaste.^30^ As the formation dental caries is influenced by multiple factors,^16^ a variety of preventive measures should be taken to control the occurrence and development of ECC. A clinical study showed that various measures were useful for the prevention of ECC in preschool children, including oral health education, topical fluoride application, the use of fluoride toothpaste, and so on.^10,18,23,26^ So far, preventive strategies have focused mainly on one age group of children and consisted of a single preventive strategy at one time. The present study we have established is a set of oral comprehensive healthcare measures, including oral health education, topical fluoride application and dental treatment, and we have assessed the effect of these measures on the prevalence of ECC in urban China.

## Materials and Methods

### Participation

The study was designed as a single-blind clinical. The eligibility criteria of the samples were as follows: children aged 4–5 years old enrolled in middle classes of kindergartens in Qingdao, China. The included children in this study were from six kindergartens in Qingdao, China with their parents providing written, informed consent. The six kindergartens were comparable in size, organisation, class and living conditions, as well as oral hygiene conditions. A random sampling method was adopted for selecting three kindergartens as the experimental group (187 children), and the remaining three kindergartens as the control group (211 children). Children with systemic or mental illness, or obvious oral infections were excluded. There was no statistically significant difference in age and gender distribution between the two groups (p >0.05) (Tables 1 and 2). The parents of the children in the experimental group voluntarily signed an informed consent form. The study was approved by the Institutional Review Board and the Ethical Committee of the Affiliated Hospital of Qingdao University.

**Table 1:** Comparison of the age between the experimental group and the control group at baseline

	Experimental group		Control group			
	M	SD	M	SD	*t*	*p*
Age	4.63	0.485	4.63	0.485	0.02	0.999 *

Independent samples t test; M, mean; SD, standard deviation. * p >0.05.

**Table 2:** Comparison of the gender between the experimental group and the control group at baseline

	Experimental group	Control group
Boys	100	112
Girls	87	99
*χ²*	0.06	
*p*	0.093 *	

2 × 2 fourfold table *χ*^2^ test. * p >0.05.

Comprehensive oral healthcare measures were provided to study the subjects in the experimental group, including oral health examination, oral health education, topical fluoride application and dental treatment. The control group received oral health examinations only twice a year. At the end of the trial, children in the control group were given the same comprehensive oral care measures as the experimental group after their parents signed informed consent forms.

### Inspection Methods and Diagnostic Criteria

Oral examinations were performed on two groups of children before and after oral education. Visual diagnosis of dental caries was performed after drying and cleaning the teeth with sterile gauze. The examination was conducted under the artificial light source by means of visual examination in combination with exploration probing, using the mouth mirror and the community periodontal index probe. The diagnosis of ECC was based on the diagnostic criteria of WHO.^20^ The decayed teeth (dt), decayed tooth surfaces (ds), missing teeth (mt), missing tooth surfaces (ms), filling teeth (ft) and filled tooth surfaces (fs) were recorded, respectively. On the day of the examination, parents were given information about the oral condition of their child.

### Quality Control

Prior to the oral examination, diagnostic criteria of ECC were standardised among examiners to reduce deviations among examiners. A total of four dentists were then selected. Standardised training of oral examination methods and recording methods was carried out by senior doctors.^4^ examiners subsequently underwent standard conformance tests, and the kappa values were greater than 0.8. Quality control was carried out by a senior doctor during the examination by interrater reliability. Lastly, the two examinations of the six kindergartens were completed by the same group of doctors.

### Comprehensive Oral Healthcare Measures

Comprehensive oral healthcare measures included oral health examination, topical fluoride application, oral health education and dental treatment. After the first oral examination in the two groups of children, the children in the experimental group were given comprehensive oral healthcare measures, including topical fluoride application twice a year, regular oral health education and dental treatment.

Acid phosphate fluoride (APF) is an effective anticaries agent that has been widely studied and applied in the past half century. In this study, we used APF foam (Laclede Foam, 1.23% APF, pH 3.5, USA), a new form of application relative to APF gel,^28^ for caries prevention. Before applying fluoride, we selected a suitably sized disposable tray and dried the tooth with sterile gauze. The fluoride foam was placed in the tray and the volume was not more than 40%. The tray was then placed on the child’s teeth, indicating the maxillary and mandibular teeth of the child to bite. The tray was put into the child’s mouth for 4 min, and the child informed not to swallow until the tray was removed.^12^ Meanwhile, the teacher was told that the child was not allowed to gargle, drink or eat for at least 30 min. These children received a total of two APF foam antifluoride treatments once every 6 months.^27^ The procedure was performed by professionally trained dentists.

Before the intervention, a PowerPoint (PPT) presentation covering the standardised oral health education was given to kindergarten teachers in the experimental group by a professional dentist. A set of PPT slides and animated video were specially made for the trained teachers to teach the children. Physicians and kindergarten teachers gave a 20-min oral health lesson monthly to children in the experimental group, 20 min each time. The focus of education included: (1) the correct way to brush teeth; (2) tooth decay hazards in deciduous teeth;(3) the relationship between sweets and caries. At the same time, parents in the experimental group received an oral health education lecture every 3 months. In order to ensure that the parents whose child received intervention can receive lectures on oral health education, and to coordinate the parents’ time, we provided a week-long lecture with the same content, until all the parents were involved in the lecture. The focus of oral hygiene education lecture for parents were: development of deciduous teeth and permanent teeth in embryonic period; the importance of deciduous teeth; the occurrence and development of dental caries; the importance of brushing teeth and the timing of brushing teeth; the correct way to brush teeth; harm of caries in deciduous teeth; the importance of regular oral examinations for children; the relationship between sweets and caries; parents are the best teachers for children; correct oral healthcare concept, and so on. The experimental kindergartens held a brushing teeth competition once a month to test the results of brushing teeth. During the event, oral health knowledge brochures and other promotional materials were distributed to parents. The Affiliated Hospital of Qingdao University was responsible for this study. Under the premise that the parents volunteered to take children to come to the hospital, the free dental treatment was provided for children in the experimental group.

### Baseline Assessment

The baseline assessment included children’s gender and age, oral examination and evaluation of oral questionnaire. Before the comprehensive oral healthcare, a questionnaire was issued to parents of children under the coordination of doctors and teachers. The questionnaire was designed and completed based on a child caries survey study and a cross-sectional study.^17^ The questionnaire included assessing risk factors of oral problems, children’s eating habits, children’s oral hygiene habits, parents’ oral hygiene habits and their awareness of oral healthcare in children.

To determine the children’s oral hygiene habits and eating habits, we surveyed the following:

- The frequency of eating sweets and drinks every day
- The number of times that teeth were brushed every day
- The amount of time teeth were brushed each time brushing of teeth occurred
- The number of times that daily mouthwash was used after meals
- The number of times for regular oral examinations for children

Information regarding parents’ oral hygiene habits and the care and protection of children’s oral hygiene included:

- The number of times that parents helped children brush their teeth
- The number of times that parents brushed their teeth
- The condition that parents monitor children to brush their teeth

The questionnaire was prepared in the format of multiple-choice questions. The questionnaires were distributed to parents of children by kindergarten teachers and dental examiners during the first oral examination. Parents filled out the questionnaire and returned it at the end of the examination to avoid missing information and guarantee the authenticity and accuracy of the data. The questionnaire directly reflected the parents’ recognition and attitude towards oral healthcare, as well as the oral hygiene habits of the children on weekdays.

### Oral Comprehensive Evaluation Survey

After 1 year’s oral comprehensive healthcare, the effect of the oral comprehensive care program was evaluated through surveys. The survey included the oral health status of two groups of children by the oral examination and the awareness and attitude of parents to children’s oral health evaluated by the oral health questionnaire.

### Statistical Analysis

All the data were analysed by the Statistical Product and Service Solutions 18.0 (SPSS, Chicago, IL, USA) according to schedule. Independent samples t test was performed on the age of two groups, and 2 × 2 fourfold table c^2^ test was performed on the sex of two groups. Independent samples t test was used to compare the differences of caries index (dt, mt, ft, dmft, ds, ms, fs and dmfs) at baseline and after 1 year between the experimental group and the control group. Paired t test was used to test the difference of each index changes before and after the intervention. The oral hygiene habits and dietary habits of two groups of children and their parents before and after intervention were tested by Wilcoxon paired symbol rank sum. p <0.05 was thought to have a statistically significant difference.

## Results

### General Information

In the baseline survey, a total of 398 children aged 4–5 years and diagnosed as ECC were enrolled in the investigation. No child dropped out during the 1-year study. There was no statistically significant difference in age between the experimental group and the control group (p >0.05, Table 1). There was no statistically significant difference in gender between the two groups (p >0.05) (Table 2).

### The Oral Condition of Children

There were no statistically significant differences in the caries index (dt, mt, ft, dmft, ds, ms, fs and dmfs) between the experimental group and the control group at baseline (all p >0.05) (Table 3). In contrast, after 1 year of oral comprehensive healthcare in the experimental group, statistically significant differences were noticed between the two groups with regard to caries index (dt, mt, ft, dmft, ds, ms, fs and dmfs) in the re assessment (all p <0.05) (Table 4).

**Table 3:** Comparison of the oral health status of the experimental group and the control group at baseline

	Experimental group		Control group		Independent test	
	M	SD	M	SD	*t*	*p*
Dt	2.48	2.99	2.33	3.07	0.51	0.613 *
Mt	0.13	0.62	0.11	0.50	0.35	0.730 *
Ft	0.09	0.41	0.11	0.48	–0.41	0.681 *
Dmft	2.59	3.27	2.48	3.33	0.33	0.740 *
Ds	2.96	3.94	2.58	3.62	1.00	0.318 *
Ms	0.57	2.82	0.45	2.09	0.49	0.622 *
Fs	0.09	0.45	0.11	0.48	–0.39	0.699 *
Dmfs	3.59	5.59	3.14	4.66	0.88	0.379 *

Independent samples t test. Dt, decayed teeth; Mt, missing teeth; Ft, filled teeth; Dmft, decayed, missing and filled teeth; Ds, decayed tooth surfaces; Ms, missing tooth surfaces; Fs, filled tooth surfaces; Dmfs: decayed, missing and filled tooth surfaces; SD, standard deviation. *: p >0.05.

**Table 4:** Comparison of the oral health status of the experimental group and the control group after a year

	Experimental group		Control group		Independent test		
	M	SD	M	SD	*t*	*p*	
Dt	2.91	2.54	3.39	3.41	–3.93	<0.001	***
Mt	0.13	0.65	0.21	0.75	–1.12	0.264	
Ft	0.61	1.32	0.21	0.87	3.61	<0.001	***
Dmft	2.94	3.37	3.81	3.89	–2.36	0.019	*
Ds	2.61	3.28	4.66	5.55	–4.42	<0.001	***
Ms	0.56	2.75	0.87	3.12	–1.05	0.295	
Fs	0.76	1.77	0.28	1.16	3.29	<0.001	***
Dmfs	3.94	5.39	5.81	7.24	–2.90	0.004	**

Independent samples t test Dt, decayed teeth; Mt, missing teeth; Ft, filled teeth; Dmft, decayed, missing and filled teeth; Ds, decayed tooth surfaces; Ms, missing tooth surfaces; Fs, filled tooth surfaces; Dmfs, decayed, missing and filled tooth surfaces; SD, standard deviation. *:0.01 <p <0.05; **:0.001< p <0.01; ***: p <0.001.

After a year of oral comprehensive healthcare, paired t tests were performed on the changes of oral indexes in two groups of children. The results showed that there were statistically significant differences in dt, ft, dmft, ds, fs and dmfs) (p <0.001), as well as in mt and ms (p <0.01; Table 5).

**Table 5:** Comparison of the variation in the oral health status between the two groups after a year

	Experimental group		Control group		Independent test		
	M	SD	M	SD	*t*	*p*	
Dt	–0.29	1.42	1.06	1.55	–0.94	<0.001	***
Mt	0.01	0.07	0.10	0.50	–2.70	0.007	**
Ft	0.52	1.17	0.10	0.63	4.51	<0.001	***
Dmft	0.35	1.23	1.32	1.75	–6.36	<0.001	***
Ds	–0.35	1.92	2.08	3.02	–9.45	<0.001	***
Ms	–0.01	0.15	0.42	2.03	–2.91	0.004	**
Fs	0.67	1.64	0.17	0.90	3.87	<0.001	***
Dmfs	0.37	1.17	2.67	3.35	–8.89	<0.001	***

Paired t test. Dt, decayed teeth; Mt, missing teeth; Ft, filled teeth; Dmft decayed, missing and filled teeth; Ds, decayed tooth surfaces; Ms, missing tooth surfaces; Fs, filled tooth surfaces; Dmfs, decayed, missing and filled tooth surfaces; SD, standard deviation. *:0.01< p <0.05; **:0.001< p <0.01; ***: p <0.001.

### Changes of Oral Hygiene Habits and Dietary Habits in Children and Parents

The oral hygiene habits and dietary habits of children and parents in the experimental group were compared with those in the control group after a year of oral comprehensive healthcare. The results showed statistically significant progress in the experimental group according to the above content (all p <0.001) (Table 6). Overall, the experimental group of children and parents had better oral hygiene habits and eating habits than the control group. The ratio of children’s oral hygiene and dietary habits to parents’ oral hygiene habits were also significantly improved after a year (Table 7).

**Table 6:** Comparison of two groups of children and parents after 1 year of oral hygiene habits

Experimental control group	Test group	Independent test		
		W	Z	p
The children	The frequency of eating sweets and drinks every day	36245.5	–6.37	<0.001 *
	The number of times of brushing teeth every day	24379.5	–12.30	<0.001 *
	The time of brushing teeth each time	28737.0	–12.76	<0.001 *
	The number of times of daily mouthwash after meals	23993.5	–12.40	<0.001 *
	The time for regular oral examinations for children	32302.0	–8.91	<0.001 *
The parents	The number of times of brushing teeth every day	31747.5	–5.63	<0.001 *
	Parents supervise their children to brush their teeth	27398.0	–9.33	<0.001 *
	Parents help their children brush their teeth	25005.0	–11.90	<0.001 *

Wilcoxon rank sum test; W, Wilcoxon W; *: p <0.001.

**Table 7:** Comparison of two groups of children and parents after 1 year of oral hygiene habits

		Experimental group		Control group	
		Baseline	Evaluation	Baseline	Evaluation
		N (%)	N (%)	N (%)	N (%)
The children	Eat sweets more than twice a day	2 (0.15)	6 (0.032)	21 (0.10)	28 (0.133)
	Brush their teeth twice a day	16 (0.086)	137 (0.733)	25 (0.118)	27 (0.128)
	Brush their teeth every time at least 3 min	16 (0.086)	147 (0.786)	16 (0.076)	32 (0.152)
	Rinse mouth more than twice a day after a meal	18 (0.096)	142 (0.759)	29 (0.137)	39 (0.185)
	Regular oral examination	9 (0.048)	41 (0.219)	10 (0.047)	14 (0.066)
The parents	Brush their teeth twice a day	65 (0.348)	136 (0.727)	82 (0.389)	97 (0.46)
	Supervise their child every day to brush your teeth	79 (0.422)	126 (0.634)	64 (0.303)	61 (0.289)
	Often help their children brush their teeth	18 (0.096)	143 (0.765)	29 (0.137)	34 (0.161)

N (%): Number (percentage).

## Discussion

Dental caries does great harm to the oral health of preschool children. Because the pulp horn of deciduous teeth is high, the pulp cavity is large and the root canals are rough. Once the caries involves the pulp cavity, the pulp disease will develop rapidly and form periapical lesions,^26^ which will cause swelling and pain in the periapical tissue. Recent studies^1,14^ have suggested that the risk factors of ECC include diet, bacteria and host susceptibility. Studies show oral hygiene habits, dietary habits and dental care services play an important role in the incidence of dental caries.^13,15,25^ Additionally, oral comprehensive healthcare can prevent and reduce the incidence of ECC, and the oral health awareness and attitude of the parents can be significantly improved after oral education.^23^ As a result, preschool children’s oral health education and health promotion had been proposed to be mandatory. The present study was based on the research of Rong WS, Si Y and other scholars.^21,24^ It is an effective measure to urge the preschool children to develop good oral hygiene behaviour, so as to improve the caries status of children. Although studies have reduced ECC^8,11^ by means of a topical fluoride treatment intervention, there has also been an intervention in education to reduce ECC, as has been reported in previous studies.^3^ ECC is a multifactorial disease that cannot be effectively controlled by a single method and requires a comprehensive prevention strategy. In the present study, through the establishment of a comprehensive oral healthcare model including oral health examination, oral health education, topical fluoride application and dental treatment, the caries formation in children of the experimental group was intervened. Our method not only protected children from cavities, but also prevented the development of decayed teeth by coating fluoride and filling, which allows parents, children, teachers and dentists to work together to help children improve their oral health.

The comprehensive oral healthcare model combined the first-, second- and third-grade prevention methods of dental caries. The first-grade prevention included the oral health education for parents, children and kindergarten teachers and topical fluoride application in children. The second-grade prevention additionally included the establishment of regular oral examinations. The third-grade treatments additionally included dental treatment. The results of the present study show that there was no statistically significant difference in dmft and other indexes between the experimental and control groups before the oral comprehensive healthcare. One year after the application of the comprehensive oral healthcare measures in the experimental group, dmft and other indexes of the two groups had significantly changed. However, mt and ms data showed no statistically significant differences. This was probably because the experimental period was 1 year, so the caries in children’s primary teeth had not dropped off enough within a year despite the rapid progress of dental caries. In addition, the oral hygiene habits and dietary habits of children and parents between the two groups were also significantly different from 1 year after the oral comprehensive healthcare (all p <0.001). Overall, the findings suggested that this comprehensive oral healthcare model had a positive impact on reducing ECC.

In the control group, at the end of the first oral examination, some parents were aware of the seriousness of their children’s oral problems, and took their children to the hospital to fill the teeth. As these parents and children did not receive comprehensive oral healthcare measures including oral health education and topical fluoride application, the surface of the decayed tooth of the children had been increasing in the previous year, though the number of dental caries increased. The results of the control group showed that just filling early childhood caries cannot prevent the growth of ECC in children and will not reduce the incidence of ECC. Therefore, only comprehensive oral healthcare accepted by parents and kindergartens will protect the oral health of children.

There are several limitations to this study. Firstly, the study lasted only 1 year, and it can take longer to evaluate the effectiveness of an oral promotion model. Secondly, the validity of the model could also be assessed by the indicators at microbial level. At the same time, better methods are expected to join the oral health promotion to reduce ECC in the future.

## Conclusion

Oral comprehensive healthcare for preschool children, including oral health examination, fluoride application and dental treatment, can improve awareness of oral healthcare in children and their guardians, help to develop good oral hygiene habits, improve children’s oral health status, reduce the occurrence and development of ECC in the population, and fundamentally improve the prevalence of caries in young children. Therefore, a comprehensive oral health promotion model should be recognised by teachers and parents as the oral health status of young children has become a societal concern. Our model can be readily applied to kindergartens and schools to increase oral health awareness among parents and teachers.
